# Nanoparticle delivery of AMPK activator 991 prevents its toxicity and improves muscle homeostasis in Duchenne muscular dystrophy

**DOI:** 10.1016/j.omtm.2025.101564

**Published:** 2025-08-14

**Authors:** Ilaria Andreana, Ananga Ghosh, Mathieu Repellin, Anita Kneppers, Sabrina Ben Larbi, Federica Tifni, Aurélie Fessard, Marion Martin, Jacqueline Sidi-Boumedine, David Kryza, Barbara Stella, Silvia Arpicco, Claire Bordes, Yves Chevalier, Julien Gondin, Bénédicte Chazaud, Rémi Mounier, Giovanna Lollo, Gaëtan Juban

**Affiliations:** 1 Dipartimento di Scienza e Tecnologia del Farmaco, Università di Torino, Torino, Italy; 2 Laboratoire d’Automatique, de Génie des Procédés et de Génie Pharmaceutique, Université Claude Bernard Lyon 1, CNRS UMR 5007, Villeurbanne, France; 3 Institut NeuroMyoGène, Physiopathologie et Génétique du Neurone et du Muscle, Université Claude Bernard Lyon 1, Inserm U1315, CNRS UMR 5261, Université de Lyon, Lyon, France; 4 Hospices Civils de Lyon, 69437 Lyon, France; 5 Imthernat Plateform, Centre Léon Bérard, Lyon, France; 6 Institut Universitaire de France (IUF), Paris, France

**Keywords:** Duchenne muscular dystrophy, chronic inflammation, fibrosis, AMPK, PLGA nanoparticles

## Abstract

Muscular dystrophies, such as Duchenne muscular dystrophy (DMD), are caused by permanent muscle injuries leading to chronic inflammation, with macrophages harboring an altered inflammatory profile contributing to fibrosis through the secretion of transforming growth factor β1 (TGF-β1). We previously showed that AMP-activated protein kinase (AMPK) activation reduces TGF-β1 secretion by macrophages and improves muscle homeostasis and muscle force in a DMD mouse model. However, direct AMPK activators like compound 991 show strong adverse effects *in vivo*. To overcome this toxicity, we encapsulated 991 into biodegradable polymeric poly(lactic-*co*-glycolic) acid (PLGA) nanoparticles for *in vivo* delivery. We show that 991-loaded PLGA nanoparticles retained drug activity on fibrotic macrophages *in vitro* and *in vivo*. In the D2-mdx DMD mouse model, intravenously injected PLGA nanoparticles reached macrophages in *gastrocnemius* and diaphragm muscles, two severely affected muscles in this model, but not in heart and quadriceps. Chronic intravenous injections of 991-loaded PLGA nanoparticles decreased inflammation in both *gastrocnemius* and diaphragm, which was associated with TGF-β1 level and fibrosis reduction and increase in myofiber size and muscle mass in the *gastrocnemius*, without toxicity. These results demonstrate that nanomedicine is an efficient strategy to deliver AMPK activators *in vivo* to target inflammation and improve the dystrophic muscle phenotype in the *gastrocnemius*.

## Graphical abstract

**Figure:**
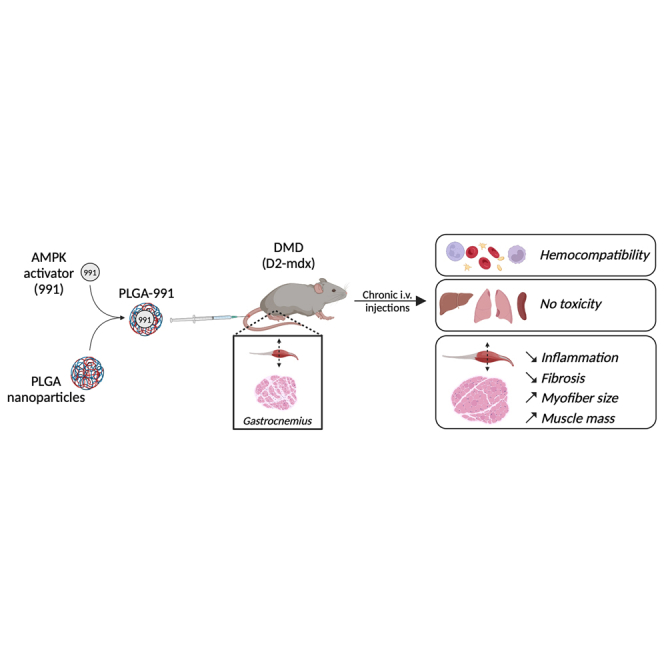

## Teaser

Duchenne muscular dystrophy (DMD) is a degenerative muscle disease associated with chronic inflammation and fibrosis. Juban and colleagues show that the encapsulation of the AMPK activator 991 into PLGA nanoparticles prevents its adverse effects and allows its delivery in muscle macrophages, improving muscle homeostasis in a DMD mouse model.

## Introduction

Duchenne muscular dystrophy (DMD) is a severe and progressive muscle-wasting disease leading to loss of ambulation, need for mechanical respiratory assistance, and premature death at around age 30 years.^1^^,^^2^ Mutations observed in DMD induce a lack of the dystrophin muscle isoform,^1^^,^^2^ which belongs to the dystroglycan complex stabilizing the link between the cytoskeleton and the sarcolemma,^3^ leading to myofiber fragility, permanent asynchronous muscle injuries, and attempts of regeneration driving chronic inflammation and fibrosis. Fibrosis is a critical component of the disease as there is a strong correlation between the level of muscle fibrosis and the loss of muscle function.^4^ Currently, there are no curative treatments for DMD, and most of the research is directed toward the development of gene therapy strategies to rescue the expression of dystrophin in patients.^5^ Standard palliative care for patients consists in the chronic administration of glucocorticoids. Although they have been shown to delay disease progression and ambulation loss,^6^ long-term exposure to glucocorticoids is associated with strong adverse effects including obesity, metabolic dysregulation, and myofiber atrophy.^7^^,^^8^ As an alternative strategy, activation of the metabolic sensor AMPK has been used to modulate muscle biology^9^^,^^10^^,^^11^^,^^12^ and ameliorate muscle dystrophic phenotype in mouse models of DMD.^13^^,^^14^^,^^15^^,^^16^^,^^17^^,^^18^ Mechanistically, AMPK activation has been shown to increase utrophin protein level,^13^^,^^14^^,^^16^^,^^18^ a functional homolog of dystrophin, stimulate autophagy,^15^^,^^18^ and trigger mitochondria biogenesis and a myogenic oxidative program.^12^^,^^13^^,^^14^^,^^16^^,^^18^

The chronic inflammation observed in DMD is characterized by the presence of pro-inflammatory and pro-fibrotic macrophages that differ from those observed after an acute damage. In normal skeletal muscle, the damage triggers blood monocyte entry into the injured area where they become pro-inflammatory macrophages, favors muscle stem cell (MuSC) proliferation,^19^^,^^20^ and limits fibro/adipogenic progenitor (FAP) expansion.^21^^,^^22^ The resolution of inflammation is triggered by their switch toward an anti-inflammatory/restorative profile,^19^ which stimulates MuSC differentiation and fusion,^20^ favors extracellular matrix (ECM) production by fibroblastic cells,^21^^,^^22^ and promotes angiogenesis.^23^ We identified that activation of the metabolic sensor AMPK is required for the macrophage inflammatory switch.^24^^,^^25^^,^^26^ In DMD, pro-inflammatory macrophages are associated with fibrosis while anti-inflammatory macrophages are associated with myogenesis.^22^^,^^27^ In this context, pharmacological skewing of pro-inflammatory/pro-fibrotic macrophages toward a pro-resolutive phenotype using indirect activators of AMPK such as metformin^22^ or sodium hydrosulfide (NaHS)^28^ reduces muscle fibrosis and improves dystrophic muscle homeostasis and function *in vivo* in a DMD mouse model, identifying AMPK as a potential therapeutic target.

In the perspective of targeting AMPK, the use of a direct activator appears to be more relevant as indirect activators may also activate other effectors, potentially leading to uncontrolled adverse effects. However, with the notable exceptions of the O304^29^ and PXL770^30^ compounds, direct AMPK activators performed poorly in clinical trials either because of a lack of patient condition improvement or because of adverse effects.^31^^,^^32^ Among them, the synthetic compound 991 (also known as Ex229) is a cyclic benzimidazole derivative identified as a potent allosteric activator of AMPK in skeletal muscle *ex vivo*^33^ that inhibits TGF-β secretion by fibrotic macrophages *in vitro.*^22^ Therefore, it represents a good candidate to selectively and potently activate AMPK in DMD muscle to modulate inflammation, dampen fibrosis, and improve muscle function. While the literature does not report any study describing the use of 991 *in vivo*, it is accepted that 991 injection in mice induces strong adverse effects such as hemolysis, platelet aggregation, and liver alteration (unpublished observations), preventing its direct use.

Recently, gold^34^ or mesoporous silica^35^ nanoparticles (NPs) have been used to target muscles in dystrophic mouse models. However, chronic administration of these types of NPs can be associated with toxic effects.^36^^,^^37^ To enable the AMPK activator 991 delivery *in vivo,* we optimized its encapsulation into biodegradable polymeric poly(lactic-*co*-glycolic) acid (PLGA) NPs. PLGA NPs exhibit a small size (<100 nm) that allows them to enter passively and accumulate into inflamed tissues through capillaries, thanks to the enhanced permeation and retention (EPR) effect.^38^ Moreover, PLGA is Food and Drug Administration (FDA) and European Medicines Agency (EMA) approved for intravenous (i.v.) administration and has already been used in the form of NPs in clinical trials for the delivery of anti-cancer treatments.^39^ When inside cells, PLGA undergoes hydrolytic degradation of the backbone ester groups, releasing its content and producing lactic and glycolic acid, which are metabolized through natural cellular pathways as Krebs cycle.^40^ In the context of DMD, systemic/i.v. administration appears to be the most relevant as it potentially allows to target all the affected muscles throughout the body.

Here, we investigated the possibility to deliver the AMPK activator 991 *in vivo* through its encapsulation into PLGA NPs in the D2-mdx mouse model, which exhibits a severe dystrophy.^41^^,^^42^^,^^43^ We first assessed the feasibility of encapsulating 991 into PLGA NPs *via* a continuous scalable process, namely microfluidics, to obtain a reproducible and highly efficient incorporation of the drug in the NPs while guaranteeing the automation and continuous production of NP formation. Moreover, we produced a dry form of the loaded-PLGA NPs to increase their storage stability over 21 days and reach a complete elimination of residual organic solvents. We next showed that 991 retained its biological activity toward fibrotic macrophages *in vitro* and *in vivo* in the *gastrocnemius* when loaded into PLGA NPs. Then, we demonstrated that upon i.v. injection, PLGA NPs reached both *gastrocnemius* and diaphragm muscles of the D2-mdx mouse where they are internalized by macrophages. Finally, chronic i.v. injections of 991-loaded PLGA NPs decreased the number of macrophages and the level of the pro-fibrotic cytokine TGF-β1 in the *gastrocnemius*, which was associated with improved muscle homeostasis in this muscle without inducing toxicity.

## Results

### Chronic 991 treatment shows toxicity *in vivo*

In order to evaluate its efficiency to improve DMD muscle homeostasis, compound 991 was administrated by chronic (every other day) i.v. injections (Figure S1A). The treatment was stopped after 2 weeks because of tail edema and necrosis. Although no impact of 991 treatment was observed on platelets and leukocytes, the number of circulating erythrocytes showed a dramatic decrease (−40% and −29% vs. PBS and Diluent, respectively) (Figure S1B). Similarly, a strong increase in alanine transaminase (ALT) (+900% and +97% vs. PBS and diluent, respectively) was observed in 991-treated mouse plasma (Figure S1C), indicative of liver damage.

These results show that 991 administration *in vivo* is associated with serious adverse effects.

### Optimization of 991-loaded PLGA nanoparticles by continuous formulation process using DoE approach

**Figure 1:**
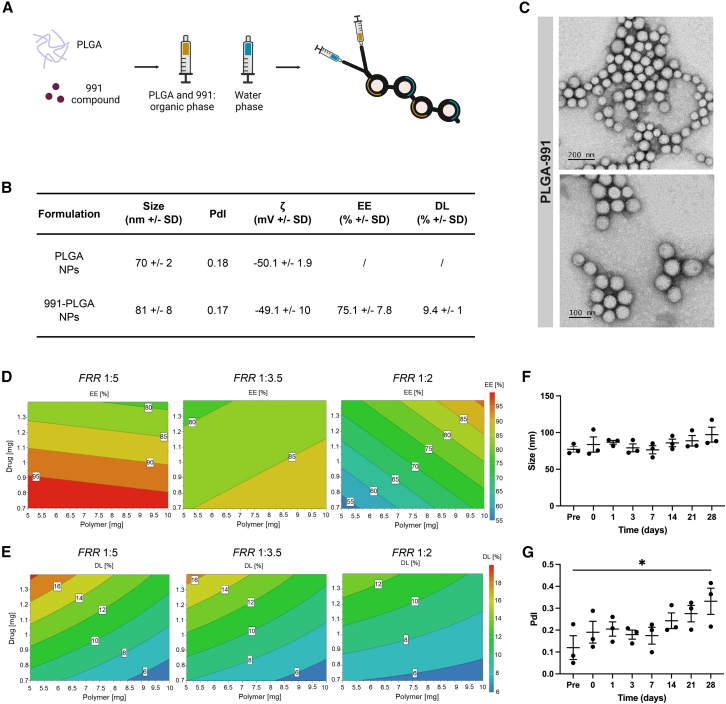
Manufacturing translation of 991-loaded PLGA NPs: formulative optimization of a continuous production process (A) Schematic representation of the microfluidic micromixing. (B) Polymer NP physicochemical analysis. Values are given as mean ± SD (*n* = 3). (C) TTEM of empty PLGA NPs (top) or 991-loaded PLGA NPs (bottom). (D and E) Contour plots of the response surface for EE (D) and DL (E) at FRR 1:5 (left), 1:3.5 (middle), and 1:2 (right). (F and G) Characterization of 991-loaded NPs after freeze-drying. Analysis of the size (F) and PdI (G) upon resuspension of loaded freeze-dried NPs. Results are shown as mean ± SEM of *n* = 3 experiments. ∗*p* < 0.05 by one-way ANOVA with Tukey's multiple comparison correction.

In an attempt to overcome 991 toxic effects, we encapsulated it into PLGA NPs. NPs were successfully formulated by both conventional manufacturing method (nanoprecipitation) and microfluidic technique (Table S1). Based on the data obtained by nanoprecipitation (NPs in the range of 150–170 nm, polydispersity index [PdI] in the range of 0.05–0.18, and high encapsulation efficiency [EE] of 85%), we produced PLGA NPs using a microfluidic system to benefit from controlled flow condition and the efficient mixing at high flow rates of toroidal micromixer (Figure 1A). Polymer nanocarriers were prepared by setting a total flow rate (TFR) of 12 mL/min and a flow rate ratio (FRR) of 1:2 to obtain sub-100 nm NPs (Figure 1B). In the controlled mixing process, NPs were characterized by a size of 70 and 81 nm, for empty and loaded nanosystems, respectively, with a slight diameter change in the presence of the encapsulated drug. Furthermore, transmission electron microscopy (TEM) confirmed the sub-100 nm mean diameter and highlighted the rounded shape of microfluidic-prepared NPs (Figure 1C). PLGA NPs are widely described in the literature for their ability to encapsulate various drug molecules.^44^^,^^45^ However, the impacts of FRR, polymer, and drug concentration, in this case 991 concentration, were investigated for the microfluidic optimization to define a roadmap for a continuous production of NPs via design of experiment (DoE) approach. EE and drug loading (DL) were identified as critical quality attributes that are related to quality product. Using a TFR of 12 mL/min, we obtained NPs characterized by a mean diameter below 150 nm and a PdI around 0.2 (Table S2), as shown by previous results of polymer NP preparation at high TFR (10–15 mL/min).^46^^,^^47^ The fitting quality of the developed models for EE and DL was confirmed by the determination coefficients R^2^ and the residual error value of each experiment, within the range of 2 × SD_exp_ (Table S3). The results that we obtained from the DoE evidenced the influence of operative and formulative parameters on the size, EE, and drug-loaded PLGA NPs. Regarding the EE (Figure 1D), the DoE results showed that it is maximized at low polymer concentration (5 mg/mL) since the amount of polymer is not enough to entrap the drug in a high amount. As expected, DL increased with the drug concentration and by decreasing the polymer amount. DL was slightly more influenced by the drug amount than the polymer concentration (Figure 1E).

The best compromise in terms of EE and DL was obtained by working between FRR 1:2 and 1:3.5 (experiments 8, 9, and 10) (Table S2). Thus, keeping satisfying results for EE and DL, we decided to investigate the biological activity of the formulation prepared at FRR 1:2 and with 1 mg/mL of 991 and a low PLGA concentration of 6 mg/mL that allows reducing NP mean diameter.

For deeper consideration about the pharmaceutical application of sub-100-nm-loaded PLGA NPs, NMR analysis was performed to evaluate the presence of residual organic solvents, which could impact the stability and biocompatibility of PLGA formulation. 1H NMR spectra analysis showed residual of acetonitrile even after NP purification after dialysis (data not shown). For this reason and to obtain a dry form of NPs improving their storage stability, the formulation was freeze-dried after solvent elimination in the presence of 1%–12% (w/v) of trehalose as preferential cryoprotecting agent.^48^ After freeze-drying, all the loaded formulations had a non-collapsed cake aspect, allowing an easy resuspension in Milli-Q water. In addition, the 12% w/v of trehalose allowed NPs to retain the size and shape they had before freeze-drying for up to 21 days (Figures 1F, 1G, and S2) and a suitable osmolarity (286 mOsm) for *in vivo* administration (Table S4).

### PLGA encapsulation of the 991 AMPK activator retains its anti-fibrotic properties *in vitro*

**Figure 2:**
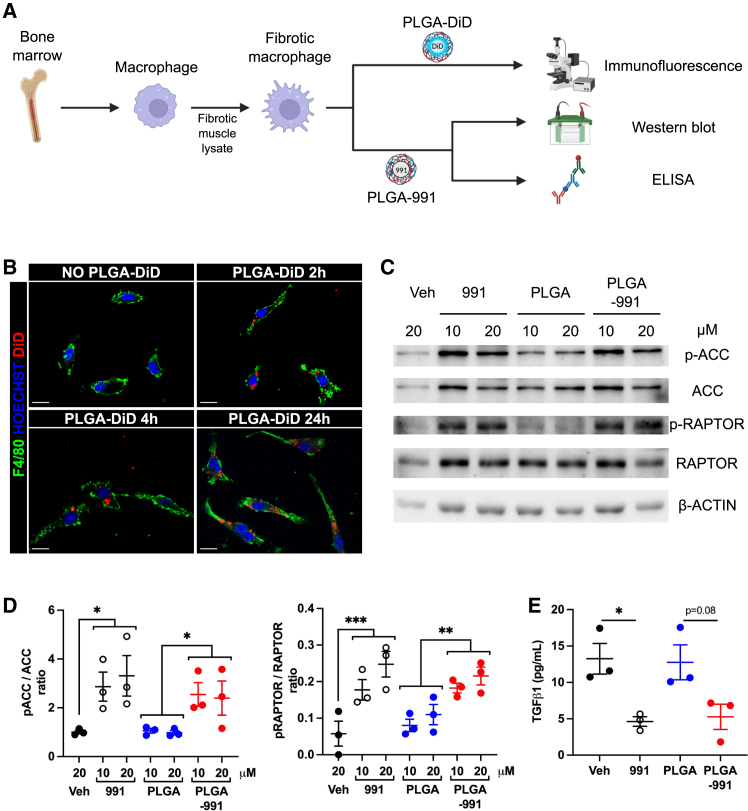
PLGA encapsulation of 991 AMPK activator retains its anti-fibrotic properties (A) BMDMs were activated into fibrotic macrophages and incubated with various types of PLGA NPs. (B) Cells were incubated without or with DiD-loaded NPs (PLGA-DiD) for 2, 4, and 24 h, immunolabeled with anti-F4/80 antibody and nuclei stained with Hoechst. A representative image of each time point is shown. Scale bars, 10 μm. (C–E) Fibrotic macrophages were treated without (Veh) or with 10 or 20 μM of 991 alone, empty PLGA, or PLGA-991 for 6 h, and the phosphorylation of ACC and RAPTOR was quantified by immunoblot. (C) Representative immunoblots. (D) Quantification of the ratio between the phosphorylated over the total ACC (left) or RAPTOR (right) proteins normalized to the β-ACTIN signal. (E) Fibrotic macrophages were treated without (Veh) or with 10 μM of 991 alone, empty PLGA, or PLGA-991 for 20 h, and the TGF-β1 production was measured by ELISA. Results are shown as mean ± SEM of *n* = 3 experiments. ∗*p* < 0.05, ∗∗*p* < 0.01, and ∗∗∗*p* < 0.001 by two-way (D) or one-way (E) ANOVA with Tukey’s multiple comparison correction. In (D), the two-way ANOVA was performed with a “drug” an a “concentration” factor. The stars show significant differences observed for the “drug” factor.

In order to evaluate the impact of NP encapsulation on 991 drug activity, we first tested the polymer nanosystem *in vitro* on bone-marrow-derived macrophages (BMDMs) that were activated into fibrotic macrophages by fibrotic muscle protein lysates^22^ (Figure 2A). BMDM incubation with PLGA NPs loaded with DiD fluorescent dye showed their fast internalization by macrophages, as soon as 2 h and that lasted up to 24 h (Figure 2B). The activity of encapsulated 991 was then assessed by monitoring the phosphorylation of the two AMPK targets acetyl-CoA carboxylase (ACC) and regulatory associated protein of MTOR (RAPTOR). As expected, BMDM treatment with 10 or 20 μM free 991 increased the phosphorylation of ACC (+185% and +230% vs. vehicle-treated control, respectively) and RAPTOR (+208% and +329% vs. vehicle-treated control, respectively) (Figures 2C and 2D). Notably, BMDMs treated with 991-loaded PLGA NPs (PLGA-991) at 10 and 20 μM showed a similar increase in the phosphorylation of ACC (+192% and +146% vs. PLGA-treated control, respectively) and RAPTOR (+128% and +96% vs. PLGA-treated control, respectively) (Figures 2C and 2D), indicative of AMPK activation. The capacity of PLGA-991 to inhibit TGF-β1 production by fibrotic BMDMs was then quantified by ELISA, since we showed that AMPK activation regulates the expression of LTBP4, which is required for TGF-β1 secretion.^22^ As we previously showed,^22^ 991 treatment inhibited the secretion of TGF-β1 by fibrotic BMDMs (−65% vs. vehicle-treated control) (Figure 2E). A similar reduction of TGF-β1 production was observed following incubation with PLGA-991 (−59% vs. PLGA-treated control) (Figure 2E). Importantly, the decrease was not due to a potential toxic effect, as PLGA-991 treatment even increased cell viability (Figure S3).

These results show that 991 encapsulation into PLGA NPs allows its internalization by fibrotic macrophages and retains its full activity.

### PLGA nanoparticles efficiently target diaphragm and *gastrocnemius* muscles in D2-mdx mice

**Figure 3:**
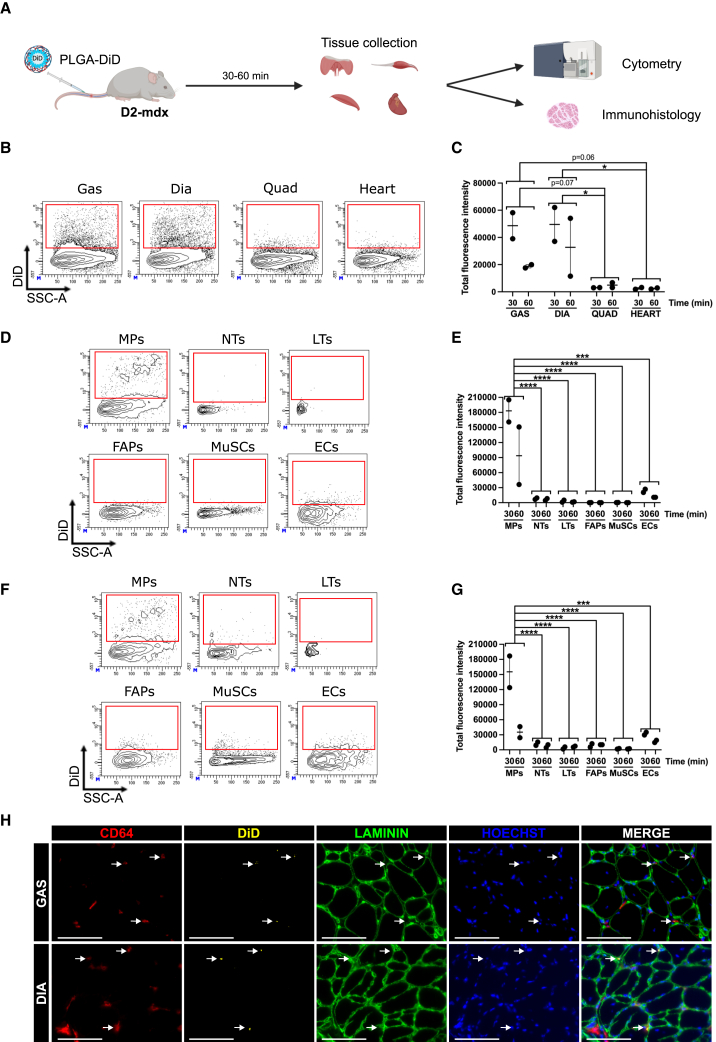
Biodistribution of PLGA NPs in D2-mdx mice (A) D2-mdx mice were injected intravenously with DiD-loaded PLGA NPs and sacrificed 30 or 60 min later, and *gastrocnemius*, diaphragm, quadriceps, and heart tissues were collected. (B–G) Tissues were dissociated, and the percentage of cells having internalized DiD-loaded PLGA NPs was determined by flow cytometry. (B and C) Flow cytometry analysis of total cells from *gastrocnemius* (Gas), diaphragm (Dia), quadriceps (Quad), and heart. (B) Representative plots. (C) Quantification of total DiD fluorescence intensity in all cells obtained by multiplying the percentage of cells labeled by DiD with DiD mean fluorescence intensity. (D and E) Flow cytometry analysis of DiD fluorescence in various cell populations from diaphragm (MPs: macrophages; NTs: neutrophils; LTs: T lymphocytes; FAPs: fibro-adipogenic progenitors; MuSCs: muscle stem cells; ECs: endothelial cells). (D) Representative plots. (E) Quantification of total DiD fluorescence intensity. (F and G) Flow cytometry analysis of DiD fluorescence in various cell populations from *gastrocnemius*. (F) Representative plots. (G) Quantification of total DiD fluorescence intensity. (H) *Gastrocnemius* (GAS, top) and diaphragm (DIA, bottom) muscle sections were immunostained for CD64 and Laminin. The white arrows show macrophages associated with PLGA NPs. Scale bars, 100 μm. Results are shown as mean ± SEM of *n* = 2 experiments. ∗*p* < 0.05, ∗∗∗*p* < 0.001, and ∗∗∗∗*p* < 0.0001 by two-way ANOVA with Tukey’s multiple comparison correction performed with a “time” and a “tissue” (C) or a “population” (E and G) factor. The stars show significant differences observed for the “tissue” or “population” factor.

In order to assess whether small-size PLGA NPs (≤100 nm) can reach and accumulate into skeletal muscles, fluorescent PLGA-DiD NPs were administered by i.v. injection into D2-mdx mice, and their biodistribution was assessed by imaging (Figure S4A). As previously described in other mouse models,^49^ we observed a rapid accumulation of PLGA NPs in the liver, spleen, and lungs (Figures S4B and S4C). In the liver, NP residence lasted over 72 h, but the fluorescence intensity decreased over time in the spleen (17% of initial fluorescence remaining after 48 h) and lungs (28% of initial fluorescence remaining after 24 h) (Figure S4C), indicative of an elimination of the nanosystem. Interestingly, fluorescence was also detected in the diaphragm as soon as 0.5 h, although at a lower level as compared with the other organs, with an almost complete clearance after 24 h (Figures S4B and S4D). To more accurately quantify NP uptake, flow cytometry was performed on diaphragm, as well as on *gastrocnemius*, quadriceps, and heart muscles, which exhibit high levels of inflammation and fibrosis^41^^,^^42^^,^^43^ (Figure 3A) (gating shown in Figure S4E). Interestingly, *gastrocnemius* and diaphragm muscles showed a high level of NP accumulation 30 min after administration, whereas almost no NPs were detected in the quadriceps and heart muscles (48,600 and 49,598 total fluorescence intensity in *gastrocnemius* and diaphragm vs. 3,071 and 2,528 in quadriceps and heart, respectively) (Figures 3B and 3C). Importantly, NP signal decreased at 60 min post-injection (19,960 and 32,780 total fluorescence intensity in *gastrocnemius* and diaphragm, respectively) (Figures 3B and 3C), indicating NP metabolization. At the cellular level, most of the NPs were internalized by macrophages in both the diaphragm (183,128 total fluorescence intensity in macrophages vs. 133–26,998 in other cell types) (Figures 3D and 3E) and *gastrocnemius* muscles (155,348 total fluorescence intensity in macrophages vs. 1,575–29,682 in other cell types) (Figures 3F and 3G) as barely no NPs were observed in neutrophils, T lymphocytes, FAPs, MuSCs, or endothelial cells 30 min post-injection. NP fluorescence in macrophages decreased 60 min after administration (down to 35,231 and 93,725 total fluorescence intensity in *gastrocnemius* and diaphragm, respectively) (Figures 3D–3G), indicating their metabolization by these cells. Finally, immunofluorescence analysis did not show any NP accumulation into myofibers and confirmed their association with macrophages (Figure 3H).

These results show that PLGA NPs efficiently reach the diaphragm and *gastrocnemius* muscles of D2-mdx mice where they are internalized mainly by macrophages.

### Chronic PLGA-991 treatment does not induce adverse effects

**Figure 4:**
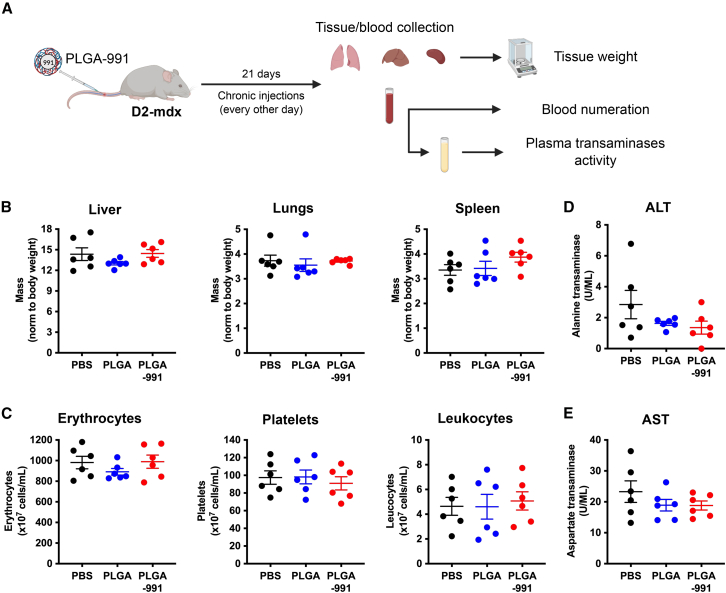
Chronic PLGA-991 treatment does not induce adverse effects (A) D2-mdx mice were treated with chronic intravenous injections of PBS, PLGA NPs, or PLGA-991 for 21 days. Liver, lungs, and spleen were harvested and blood collected. (B) Relative mass of liver, lungs, and spleen in milligrams normalized by body weight (in grams). (C) Blood cell counts showing the concentration of erythrocytes, platelets, and leukocytes. (D and E) Serum levels of alanine transaminase (ALT) (D) and aspartate transaminase (AST) (E) enzymes. Results are shown as mean ± SEM of *n* = 6 experiments. No significant difference was observed by one-way ANOVA with Tukey’s multiple comparison correction or Kruskal-Wallis test with Dunn’s multiple comparison correction (B, “Lungs”).

In order to evaluate the potential *in vivo* toxicity, PLGA-991 NPs were administrated by chronic (every other day) i.v. injections over a time period of 21 days, then blood, liver, spleen, and lungs were harvested (Figure 4A). We did not observe any effect of the PLGA-991 treatment on liver, spleen, and lung mass as compared with PBS or empty PLGA NPs (Figure 4B). Similarly, PLGA-991 chronic administration was not associated with a modification of the number of erythrocytes, platelets, and leukocytes in the blood (Figure 4C), indicative of an absence of blood toxicity. Finally, we monitored potential liver alteration by quantifying the plasma level of ALT and AST, which are two enzymes that are released in the blood upon liver injury. Importantly, neither of these enzymes were increased in the plasma of animals treated with PLGA-991 NPs as compared with PBS or empty PLGA NPs (Figures 4D and 4E).

These results show that 991 encapsulation into PLGA NPs does not induce major adverse effect on blood cells and liver homeostasis.

### Chronic PLGA-991 treatment reduces inflammation in DMD muscles and improves *gastrocnemius* muscle homeostasis in D2-mdx mice

**Figure 5:**
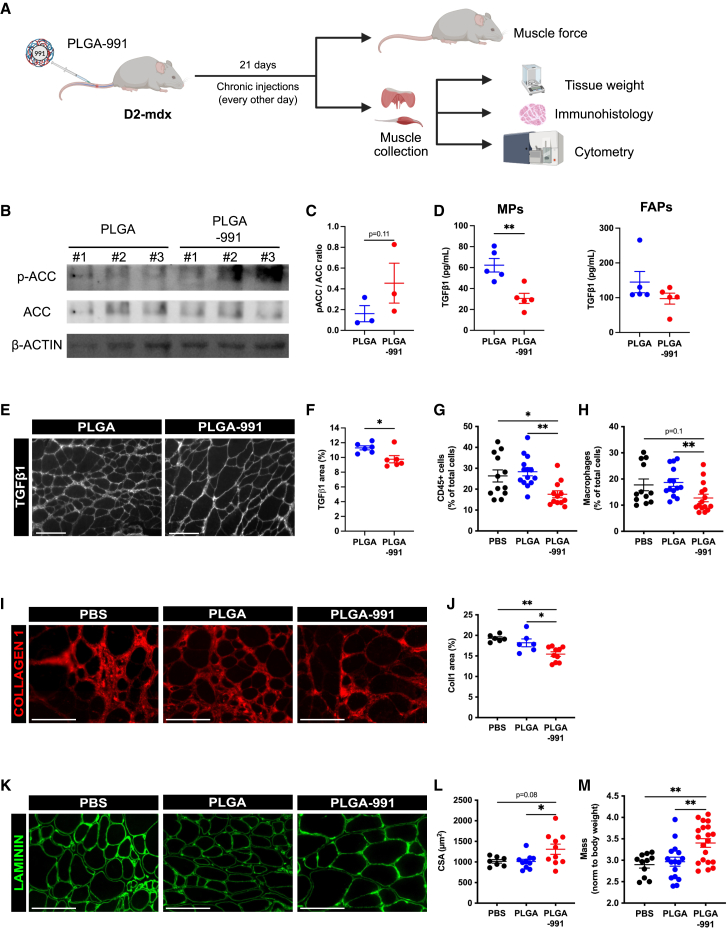
Chronic PLGA-991 treatment reduces inflammation in D2-mdx muscle and improves *Gastrocnemius* muscle homeostasis (A) D2-mdx mice were treated with chronic intravenous injections of PBS, PLGA, or PLGA-991 for 3 weeks, then *Gastrocnemius* and diaphragm muscles were harvested. (B and C) The phosphorylation of ACC was quantified by immunoblot. (B) Representative immunoblots. (C) Quantification of the ratio between the phosphorylated over the total ACC proteins normalized to the β-ACTIN signal. (D) Macrophages (MPs) and fibro-adipogenic progenitors (FAPs) were isolated from gastrocnemius, and their TGF-β1 secretion was determined by ELISA. (E and F) *Gastrocnemius* muscle sections were immunostained for TGF-β1. (E) Representative images. Scale bars, 100 μm. (F) Percentage of TGF-β1 area. (G and H) Muscles were digested, and the proportion of immune cells (CD45+) (G) and macrophages (H) was determined by flow cytometry. (I–L) *Gastrocnemius* muscle sections were immunostained for Col1 (I) and Laminin (K). Scale bars, 100 μm. (J) Percentage of Col1 area. (L) Mean myofiber cross-sectional area (CSA) determined on Laminin immunolabeling. (M) Relative *gastrocnemius* muscle mass (in milligrams) normalized by body weight (in grams). Results are shown as mean ± SEM of n = 3–12 experiments. ∗*p* < 0.05 and ∗∗*p* < 0.01 by two-tailed unpaired Student’s t test (C and D, “MPs”), two-tailed unpaired Mann-Whitney’s U test (D, “FAPs” and F), one-way ANOVA with Tukey’s multiple comparison correction (J, L, and M), or Kruskal-Wallis test with Dunn’s multiple comparison correction (G and H).

As we observed high accumulation of PLGA NPs in diaphragm and *gastrocnemius* muscles, we measured the impact of PLGA-991 treatment over a time period of 21 days (a duration at which an improvement of muscle homeostasis and function can be observed in another murine model of fibrotic DMD muscle^22^) on their homeostasis (Figure 5A). First, western bot analysis on *gastrocnemius* muscle showed a tendency toward increased phosphorylation of the AMPK target ACC in PLGA-991-treated animals (Figures 5B and 5C). Then, ELISA assay showed that macrophages isolated from *gastrocnemius* muscle from PLGA-991-treated animals secreted lower levels of TGF-β1 (Figure 5D), indicative of AMPK activation in these cells.^22^ Importantly, PLGA-991 treatment did not reduce TGF-β1 secretion by FAPs in *gastrocnemius* (Figure 5D), consistent with their lack of PLGA NPs uptake (Figures 3D–3G). At the tissue level, PLGA-991 treatment led to a lower TGF-β1 level in the *gastrocnemius* (Figures 5E and 5F; −14% vs. PLGA). Next, we analyzed the inflammation level of this muscle by flow cytometry (gating shown in Figure S5A). The number of immune cells present within the muscle tissue was reduced by PLGA-991 treatment in the gastrocnemius (−33.3% and −38% vs. PBS and empty PLGA, respectively) (Figure 5G). More precisely, this lowered inflammation was characterized by a reduction in the number of macrophages (−28.2% and −31.6% vs. PBS and empty PLGA, respectively) (Figure 5H). Notably, no significant alteration of the number of neutrophils, eosinophils, T lymphocytes, FAPs, and MuSCs was observed under PLGA-991 treatment in the gastrocnemius (Figure S5B) muscle. Of note, a tendency toward a reduction of the number of endothelial cells was observed in the *Gastrocnemius* (Figure S5B; −36% vs. PLGA).

Next, we evaluated the consequence of PLGA-991 treatment on the dystrophic phenotype. In the gastrocnemius muscle, PLGA-991 did not impact muscle damage (Figure S5C). Collagen 1 immunolabeling showed a strong reduction under PLGA-991 treatment (−20% and −15.1% vs. PBS and empty PLGA, respectively) (Figures 5I and 5J). This was associated with an increased mean cross-sectional area (CSA) of the myofibers (+29.1% and +29.6% vs. PBS and empty PLGA, respectively) (Figures 5K–5L), leading to an improved total muscle mass (+17.3% and +14.6% vs. PBS and empty PLGA, respectively) (Figure 5M). However, PLGA-991 treatment did not improve *gastrocnemius* muscle force production nor muscle fatigue (Figures S5D–S5F).

In the diaphragm, PLGA-991 treatment did not induce significant AMPK activation in the whole tissue (Figures S6A and S6B). TGF-β1 secretion was not modified in macrophages, and FAPs isolated from diaphragm (Figure S6C), as well as the TGF-β1 in the tissue (Figure S6D) of PLGA-991-treated animals. PLGA-991 treatment was associated with a lower inflammation with a reduced macrophage number (Figures S6E and S6F), with no impact on the number of neutrophils, eosinophils, T lymphocytes, FAPs, MuSCs, and endothelial cells (Figure S6G). Finally, we did not observe any improvement by PLGA-991 treatment on fibrosis (Figure S6H), myofiber CSA (Figure S6I), and muscle mass (Figure S6J).

These results show that chronic i.v. treatment with PLGA-991 dampens muscle inflammation and improves the dystrophic phenotype in the gastrocnemius muscle of D2-mdx mice.

## Discussion

In the present study, we established that the delivery of the AMPK activator 991 *in vivo* through its encapsulation into PLGA NPs is efficient in the *gastrocnemius* of the D2-mdx mouse model of DMD. As compared with mdx, D2-mdx mice exhibit a more severe dystrophic phenotype characterized by fibrosis and muscle function loss in the diaphragm and gastrocnemius muscles.^41^^,^^42^^,^^43^

Firstly, we confirmed the useful application of microfluidic in the preparation of hydrophobic-drug-loaded PLGA NPs. The translation from conventional batch mixing to microfluidic technique is a key point for achieving a more effective and easily scalable preparation method. Notably, starting from nanoprecipitation parameters, including polymer concentration, organic phase content, and aqueous/organic phase ratio, the translation into microfluidic resulted in NPs with sub-100 nm size. A DoE highlighted the crucial effects of polymer, drug concentrations, and FRR on NP features. In addition, besides their optimal pharmaceutical properties (namely biocompatibility and biodegradability), their stability and the possibility to obtain ready-to-use dry powders highlight the potential of PLGA NPs as drug delivery systems for 991.

*In vitro*, we showed that PLGA NPs were internalized by macrophages without affecting their viability, which is in accordance with previous observations.^50^ Moreover, PLGA-loaded 991 activated AMPK and inhibited TGF-β secretion by fibrotic macrophages as efficiently as the free compound, suggesting that once internalized into cells, PLGA are able to release 991.

*In vivo*, biodistribution analyses in the D2-mdx mouse model indicated a high PLGA NP uptake in the liver, spleen, and lungs as previously described in other models.^49^ Interestingly, we also observed PLGA accumulation in the diaphragm and gastrocnemius muscles with their cellular uptake and their progressive elimination from the cells, indicative of their metabolization. The lack of fluorescence detection by imaging the whole gastrocnemius muscle, due to the low sensitivity of the method, highlights the crucial need of higher sensitivity analyses such as microscopy or flow cytometry to get a more accurate view of NP distribution. The results confirmed previous analysis made with silica NPs^35^ and show that NPs passively enter into inflamed skeletal muscle in dystrophies, making NPs a vehicle of interest to target molecules into the diseased muscles of the whole body. However, PLGA NPs did not accumulate in the quadriceps and the heart, even though they show strong inflammation and fibrosis,^41^^,^^42^^,^^43^ limiting a potential clinical application at this stage. This could indicate a difference among tissues and muscles in the capacity of the macrophage populations in NP uptake, and further work will be required to characterize them in order to accurately target them. A 21-day chronic injection did not impact blood cell counts, suggesting that PLGA encapsulation of 991 prevents its uptake by blood cells and avoids hemolysis and platelet aggregation. Moreover, no increase in ALT and AST enzymes in blood was observed, indicative of a lack of liver injury, in accordance with the hypothesis of a low metabolization of PLGA NPs in the liver.^51^ In the perspective of a potential clinical application, monitoring the absence of adverse effects over a longer period of time is a prerequisite. Although we did not observe a significant AMPK activation in the whole *gastrocnemius* and diaphragm muscles, the chronic PLGA-991 treatment efficiently reduced the inflammation in both muscles, with a specific reduction in macrophage numbers. This can be explained by the fact that in these tissues, only macrophages, which represent 18% to 20% of the mononucleated cells, uptake PLGA NPs, making it difficult to detect phosphorylation of AMPK downstream targets, even if we observe a tendency toward an increased phosphorylation of the AMPK target ACC in the *gastrocnemius*. Importantly, we showed AMPK activation in macrophages from the *gastrocnemius* and the reduction of their fibrotic properties, as demonstrated by their reduced TGF-β1 secretion upon PLGA-991 treatment. On the contrary, no decrease of TGF-β1 secretion by PLGA-991 was observed in macrophages from diaphragm muscle, which was associated with no improvement of the muscle homeostasis. This difference between *gastrocnemius* and diaphragm may be due to the more severe phenotype exhibited by the diaphragm muscle, notably the higher level of fibrosis. Moreover, it suggests a different threshold of NP uptake necessary in the macrophages of these two tissues to efficiently activate AMPK and reduce their fibrotic properties. At the molecular level, we may consider that macrophages from different muscles could express different AMPK subunit isoforms. Therefore, improving NP uptake by these macrophages through NP decoration to more efficiently target them together with the characterization of AMPK isoforms to select the most relevant AMPK activator depending on the targeted muscle will be necessary to dampen their fibrotic properties and improve diaphragm homeostasis. Finally, PLGA-991 was associated with decreased fibrosis and increased myofiber size and muscle mass in the *gastrocnemius*, indicative of improved muscle homeostasis. This increase in myofiber size may be partly explained by the reduction of macrophage fibrotic properties and their acquisition of pro-myogenic capacities upon AMPK activation as previously described.^28^ However, this did not lead to increased muscle force production after 3 weeks of treatment. This suggests that the duration of the treatment may need to be extended to give more time to the muscle to convert the homeostasis amelioration (i.e., reduced fibrosis and increased myofiber size) into a functional improvement.

In conclusion, our results establish the proof of concept that the use of 991 encapsulated into PLGA NPs is safe and constitutes an efficient strategy for AMPK activator delivery into skeletal muscle in DMD to modulate inflammation and improve muscle homeostasis, which may be transposed to other direct AMPK activator to improve their efficacy and limit their potential secondary effects. Further work will be required to improve the efficacy of this strategy and broaden the spectrum of targeted muscles, in the perspective of a potential translation to a clinical application.

## Materials and methods

### Reagents

PLGA 75:25 (Resomer RG 752 H, Mw = 4–15 kDa) (analytical grade) and 378.3 g/mol D-(+)-trehalose dehydrate (trehalose) used for the freeze-drying study were purchased at Sigma-Aldrich; 991 (molar mass, Mw = 431.87 g/mol) was ordered from CliniSciences. Float-A-LyzerTM G2 Dialysis devices (3.5–5 kDa MWCO, 1 mL) and 1,1′-dioctadecyl-3,3,3′,3′-tetramethylindodicarbocyanine, 4-chlorobenzenesulfonate salt (DiD) were purchased from Fisher Scientific. Sodium silicotungstate used for staining in TEM was supplied by Agar Scientific. Ultra-pure water was obtained using a Milli Q Academic System from Merk Millipore; 991 (M_w_ = 431.87 g/mol) was obtained from SpiroChem AG and resuspended in methanol.

### Preparation of PLGA NPs by nanoprecipitation

Drug-loaded NPs were prepared by the nanoprecipitation technique.^52^ Briefly, 6 mg of PLGA were dissolved in 800 μL of acetonitrile and added with 200 μL of a methanolic stock solution of active molecule (5 mg/mL). This organic solution was then dropped into 2 mL of Milli-Q water under magnetic stirring for 30 s. Precipitation of NPs occurred spontaneously. To remove the organic solvent and purify the NPs from the non-encapsulated drug, drug-loaded NPs were dialyzed 1 h against Milli-Q water at room temperature (RT). Empty NPs, without adding drug molecule, were prepared as well. The aqueous suspension was then stored at 4°C.

### Preparation of PLGA NPs by microfluidic technique

991-loaded PLGA NPs were obtained by microfluidic technique using benchtop NanoAssemblr provided by Precision NanoSystem, Inc. Disposable cartridges with a specific toroidal micromixer, with estimated channel dimensions of 500 μm (W) and 200 μm (H), were employed together with the automated NanoAssemblr. Briefly, for each preparation, to 6 mg of PLGA 75:25 dissolved in acetonitrile, an aliquot (200 μL) of a methanolic stock solution of 991 (5 mg/mL) was added in a total volume of 1 mL. The organic phase comprised of the polymer/drug solution was injected into one port of the NanoAssemblr instrument. The aqueous phase was simultaneously injected into the second port of the system to maintain a 1:2 organic:aqueous flow rate ratio (FRR) and 12 mL·min^−1^ total flow rate (TFR). NP products were gathered in a 15 mL falcon tube, while separately disposing of the initial 0.45 mL and the final volume of 0.05 mL of NP product. To remove the organic solvent and purify the NPs from the non-incorporated drug, 991-loaded NPs were dialyzed (Spectrum Spectra/Por Float-A-Lyzer G2 Dialysis devices [M_w_ = 3.5–5 kDa]) against Milli-Q water at RT for 1 h. Unloaded (i.e., without adding 991) and DiD-loaded NPs were prepared as well. The particles were then stored at 4°C.

### Physicochemical characterization of empty and loaded NPs

The hydrodynamic diameter, polydispersity index (PdI), and surface potential of the prepared particles were analyzed by dynamic light scattering (DLS) using a Malvern Zetasizer Nano ZS instrument (Malvern Instruments S.A., Worcestershire, UK). The angle was set at 173°, and measurements were carried out at 25°C after dilution (1:10) of the particulate suspension in Milli-Q water. The zeta potential (*ζ*) was calculated from the electrophoretic mobility measured for samples diluted in KCl 1 mM. The physical stability of NP suspensions at storage conditions (4°C) was determined by evaluating at different interval times the mean diameter, the PdI, and *ζ*. Each measurement was carried out in triplicate.

### TEM

The morphology of NPs was evaluated by TEM performed with a Philips CM120 microscope at the Center Technologique des Microstructures (CTμ) of the University Lyon 1 (Villeurbanne, France). The diluted samples (10 μL) were dropped onto a 200 mesh carbon/formvar microscope grid (copper support coated with carbon), stained with a sodium silicotungstate aqueous solution, and slowly dried in the open air. The dry samples were observed by TEM under 120 kV acceleration voltage.

### Determination of encapsulation efficiency

The EE of drug-loaded PLGA NPs was determined by dissolving an aliquot of NP solution in acetonitrile to extract the encapsulated drug. The drug EE was calculated as follows:

(Equation 1) $$EE\left(\%\right)=\frac{A}{B}✕100$$

where *A* is the amount of encapsulated drug after NPs purification, and *B* is the initial amount of drug determined from a fresh aliquot of organic phase. The DL was calculated as the ratio between the amount of entrapped drug and the total nanocarrier mass x 100. The amount of incorporated drug was determined by UHPLC. NP sample was diluted with acetonitrile (CH_3_CN)/water mixture 50/50 v/v, acidified by 0.1% trifluoroacetic acid (TFA), vortexed, and filtered through 0.22 μm Nylon filters (Agilent). The UHPLC system consisted of a quaternary pump (Waters, quaternary solvent manager-R), autosampler (Waters, sample manager FTN-R), and a multiple wavelength UV detector (Waters, 2998 PDA Detector). The data were processed using Empower 3 (Waters). The analytical column was a Cortecs C18 column (50 × 4.6 mm, 2.7 μm; Waters) at 40°C; the mobile phase consisted of waters 0.1% TFA (solvent A) and CH_3_CN 0.1% TFA (solvent B) at a flow rate of 0.5 mL/min with isocratic conditions: 40% A and 60% B. The drug concentration in the column effluent was monitored by its absorbance at 254 nm. The quantification of the active molecule was done using calibration with standard solutions chromatographed under the same experimental conditions, with a concentration range of 0.25–200 μg/mL. The calibration curve was linear (*R*^2^ > 0.995).

### NP optimization using a design of experiment

A 2^3^ full factorial design was used to investigate the effect of three experimental factors (PLGA, drug concentrations, and *FRR*) and their interactions on NP physicochemical characteristics. For each factor, the experimental domain whose lower and upper limits correspond to the coded levels −1 and +1, respectively, was defined. The center point corresponds to the coded level 0. As reported in Table 1, the polymer concentration (*X*_1_) varied from 5 to 10 mg/mL, *FRR* (*X*_2_) from 1:2 to 1:5, and drug amount (*X*_3_) from 0.7 to 1.4 mg/mL. The studied responses were the EE (*Y*_1_) and the DL (*Y*_2_) as NP characteristics to be well controlled.

**Table 1:** Selected experimental factors and their coded levels in the 2^3^ full factorial design

Experimental factors	Coded levels		
	−1	0	+1
PLGA conc. (mg/mL) (*X*_1_)	5	7.5	10
*FRR* (*X*_2_)	1:5	1:3.5	1:2
Drug conc. (mg/mL) (*X*_3_)	0.7	1.1	1.4

The full factorial design consisted in 2^3^ runs defined as every possible combination of +1 and −1 levels selected in turn and allowed calculating a synergistic model, as follows:

(Equation 2) $$\hat{y_{i}}=b_{0}+b_{1}X_{1}+b_{2}X_{2}+b_{3}X_{3}+b_{12}X_{1}X_{2}+b_{13}X_{1}X_{3}+b_{23}X_{2}X_{3}$$

where $\hat{y_{i}}$ is the predicted response for experiment i, *b*_0_ is the intercept, the coefficients *b*_i_ correspond to the main effects of the experimental factors (*X*_i_), and the coefficients *b*_ij_ are the effects of the two-factor interactions.

Two center points were also added in order to estimate the variance of the experimental error and to evaluate the predictive performance of the developed models. The corresponding experimentation plan is presented in Table S2. Multiple linear regression calculations, statistical analysis, and response contour plots were performed with Umetrics MODDE 12.0 software.

### Freeze-drying of drug-loaded NPs

Drug-loaded NPs were freeze-dried using the CRYONEXT pilot freeze dryer (Cryotec). The suspension was prepared by diluting the formulation 1/5 with a solution of trehalose at a final concentration of 12% w/v. The freeze-drying process started reaching an initial cool temperature of −35°C for 3 h. Then, the temperature of the shelf was increased to −20°C for 10 h with a chamber pressure of 0.25 mBar to remove ice by sublimation (primary drying). Secondary drying was carried out for 5 h at 4°C, with 0.1 mBar pressure. Finally, freeze-dried NPs were resuspended in 1 mL of Milli-Q water and vortexed until complete dissolution of the pellet. Size, PdI, ζ, EE, morphology, and storage physical stability at 4°C of NPs were measured after resuspension. The osmolarity of 50 μL of reconstituted sample was measured at RT using a Gonotec Osmomat 3000 provided by Thermo Fisher Scientific (Waltham, USA).

### Determination and quantification of organic solvent residuals

NPs were dialyzed for 1 h against Milli-Q water. The quantifications of remaining acetonitrile were performed by ^1^H NMR analysis. ^1^H NMR spectra were recorded on the liquid samples in 5 mm tubes with a liquid-state high-resolution Bruker AVL300 NMR spectrometer operating at 300 MHz under quantitative analysis conditions. The FID was recorded during an acquisition time of 5.5 s after a 90° radiofrequency pulse (14 μs). A delay of 60 s was applied between each scan for ensuring full relaxation of the FID. Eight scans were accumulated with conventional phase cycling; 500 μL of an aqueous suspension of NPs were added to 500 μL of deuterium oxide (D_2_O) containing 2% v/v of acetone used as internal standard. Quantification of acetonitrile residuals was done from the integral of the acetonitrile peak at a chemical shift of 1.98 ppm and that of the acetone reference peak at 2.15 ppm. The quantification of acetonitrile was performed on lyophilized samples as well.

### Mice experiments

D2-mdx (D2.B10-Dmd^mdx^/J) mice were bred and used according to French legislation, and the protocols were approved by the local Ethical committee (CEEA-010) and Ministry of Research. Experiments were conducted on males of 10–18 weeks of age.

### Murine BMDM isolation and culture

Macrophages were derived from murine bone marrow precursors as previously described^24^ and were cultured in DMEM containing 20% heat-inactivated fetal bovine serum (FBS), 30% of L929 cell-line-derived conditioned medium (enriched in CSF-1), 2.5 μg/mL of fungizone, and 100 U/mL of penicillin/streptomycin for 6–7 days. Macrophages were then activated into fibrotic cells with 1 μg/mL of total protein lysate isolated from tibialis anterior of fibrotic mdx (Fib-mdx) mouse model,^53^ in DMEM containing 10% FBS for 2 days, as previously described.^22^ For viability assessment, BMDMs were labeled with Alexa Fluor 488-conjugated Annexin V (A13201, Molecular Probes) and DAPI (564907, BD Biosciences) and analyzed on a BD FACSCanto II apparatus.

### NP internalization by BMDMs

BMDMs were seeded at 54,000 cells/cm^2^ in DMEM containing 10% of FBS on a coverslip in 12-well plates. Activated fibrotic macrophages were incubated with DiD-loaded PLGA NPs at the final DiD concentration of 0.9 μg/mL for 0, 2, 4, and 24 h at 37°C. After incubation, cells were fixed for 10 min in 4% formaldehyde, permeabilized 10 min in 0.5% Triton X-100, and saturated in 2% BSA for 1 h at room temperature. They were then labeled with anti-F4/80 primary antibody (E-AB-F0995A, Elabscience, 1:200) overnight at 4°C followed by an incubation with a secondary antibody coupled with Cy3 (Jacson Immunoresearch, 1:200) for 2 h at 37°C. After nuclei staining with Hoechst 33342 (Sigma), coverslips were mounted in Fluoromount (Interchim) and imaged on a TCS SP5-X confocal microscope (Leica microsystems). Images were analyzed with Las X software (Leica Microsystems).

### Isolation of macrophages and FAPs from muscle

Macrophages were freshly isolated from gastrocnemius and diaphragm muscles using magnetic beads enrichment as previously described,^54^ except DMEM without red phenol and charcoal-stripped FBS were used. FAPs were isolated from the negative fraction by preplating method^54^ also using DMEM without red phenol and charcoal-stripped FBS.

### TGF-β1 quantification

BMDMs were seeded at 100,000 cells/cm^2^ in 48-well plates in DMEM containing 10% of FBS. One hour before treatment, culture medium was replaced by DMEM without red phenol (Gibco) supplemented with 10% charcoal-stripped FBS (Gibco), and activated fibrotic macrophages were incubated for 20 h with 10 μM 991, empty PLGA NPs, or PLGA-991. Vehicle control (Veh) consisted of a treatment with an equivalent concentration of methanol. For cells isolated from muscle, macrophages (50,000 cells/cm^2^) and FAPs (33,000 cells/cm^2^) were incubated in 48-well plates for 6 h in DMEM without red phenol containing 10% of charcoal-stripped FBS. After 2 washes with PBS, cells were cultured for 20 h in DMEM without red phenol containing 0.1% BSA. The conditioned medium was then harvested and centrifuged for 5 min at 500 g to remove cell debris. The total amount of TGF-β1 was determined on technical duplicates by ELISA (88-8350-22, Invitrogen), after heat denaturation followed by acidic treatment and neutralization according to the manufacturer’s instructions.

### Western blot

BMDMs were seeded in DMEM containing 10% of FBS at 208,000 cells/cm^2^ in 6-well plates and activated into fibrotic macrophages. One hour before treatment, culture medium was replaced by DMEM without red phenol (Gibco) supplemented with 10% charcoal-stripped FBS (Gibco), and cells were treated with 10 or 20 μM 991, empty PLGA NPs, or PLGA-991 for 6 h. Proteins were isolated in lysis buffer containing 50 mM Tris-HCL (pH 7.5), 1 mM EDTA, 1 mM EGTA, 0.27 M sucrose, 1% Triton, 20 mM glycerol-2- phosphate disodium, 50 mM NaF, 0.5 mM PMSF, 1 mM benzamidine, 1 mM Na_3_VO_4_, and 1% cocktail phosphatase inhibitor 3 (Sigma-Aldrich, P0044) for 30 min on ice and centrifuged for 10 min at 16,100 g to remove debris. Ten micrograms of proteins were subjected to SDS-PAGE and transferred onto a nitrocellulose membrane, which was probed with antibodies against p-ACC (#3661, Cell Signaling, 1:1,000), ACC (#3676, Cell Signaling, 1:1,000), p-RAPTOR (#, 1:1000), RAPTOR (#, 1:1,000), or b-actin (#A5316, Sigma-Aldrich, 1:5,000). Blots were revealed on a Chemidoc imager (Bio-Rad) using SuperSignal West Femto Maximum Sensitivity Substrate (Thermo Scientific), and signal intensity was quantified using Image Lab Software (Bio-Rad).

### Organ biodistribution

Two hundred microliters of DiD-loaded NPs were administered to anesthetized mice by i.v. injection in the tail vein. At scheduled time points (0.5, 1, 24, 48, and 72 h), anesthetized animals were fully imaged in a light-tight chamber where a controlled flow of 1.5% isoflurane in the air was administered to maintain anesthesia. Fluorescence images, as well as bright-field pictures of mice’s whole body (ventral and prone view), were acquired *via* a back-thinned CCD-cooled camera ORCA II-BT-512G (Hamamatsu Photonics) using a colored glass long-pass RG 665 filter (Melles Griot), which cuts off all excitation light. Optical excitation was carried out at 644 nm, and the emission wavelength was detected at 655 nm. At each time point, mice were sacrificed and tissues and organs (lungs, liver, spleen, diaphragm, and gastrocnemius) were harvested for *ex vivo* imaging. Images were analyzed using the Wasabi software 1.5 (Hamamatsu Photonics).

### Chronic 991 intravenous injection

991 solution diluted at 1 mg/mL in 45% Saline buffer, 10% DMSO, 40% PEG300, and 5% Tween-80 was administered every other day to anesthetized mice by i.v. injection in the tail vein (70 μL for a 20 g mouse corresponding to a dose of 3.5 mg/kg of drug). Control mice were injected similarly with the same volume of diluent only or PBS.

### Chronic PLGA-991 intravenous injection

Two hundred microliters of empty PLGA NPs or PLGA-991 were administered every other day to anesthetized mice by i.v. injection in the tail vein (3.5 mg/kg of drug). Control mice were injected similarly with the same volume of PBS.

### Blood cell count

Blood was collected by cardiac puncture directly after sacrifice and transferred into a tube previously coated with PBS containing 50 mM EDTA. Blood was diluted in PBS, and erythrocytes were counted on a hemocytometer. For platelet numeration, blood was diluted in 1% ammonium oxalate (Sigma) and incubated on a hemocytometer for 15 min in a humid chamber before counting. Leukocytes were counted on a hemocytometer after blood was diluted in Türk’s solution (Sigma).

### Plasma transaminase quantification

Blood was centrifugated at 1,500 g for 15 min, and plasma was aliquoted and stored at −80°C for further analysis. ALT (CAK1002, Cohesion Biosciences) and aspartate transaminase (CAK1004, Cohesion Biosciences) levels were determined on technical duplicates of respectively 20 and 10 μL of plasma according to manufacturer’s instructions.

### Muscle flow cytometry analysis

Muscles were dissociated and digested in DMEM F/12 medium (GIBCO) containing 10 mg/mL of collagenase B and 2.4 U/mL Dispase II (Roche Diagnostics GmBH) at 37°C for 1 h, passed through a 30-μm cell strainer, and erythrocytes were lysed using ACK lysis buffer (Lonza). Dead cells were first labeled with Ghost Dye Red 780 (13-0865-T500, Tonbo Biosciences) for 30 min at 4°C, followed by incubation with anti-mouse FcR blocking reagent (130-092-575, Miltenyi Biotec) in PBS 2% FBS for 10 min at 4°C. For immune cell analysis, cells were then stained with BV510-conjugated anti-CD45 (103138, BioLegend), PE-Cy7-conjugated anti-CD64 (139314, BioLegend), APC-conjugated (17-9668, eBioscience) or PE-conjugated anti-Ly-6G (12–9668, eBioscience), FITC-conjugated anti-CD3 (11-0032-82, eBioscience), and PE-conjugated anti-Siglec-F (552126, BD Biosciences) antibodies for 30 min at 4°C. For non-immune cell analysis, cells were stained with PE-Cy7-conjugated anti-CD45 (25–0451, eBioscience), PercP/Cy5.5-conjugated anti-Sca-1 (108124, BioLegend), eFluor450-conjugated anti-CD31 (48–0311, eBioscience), and PE-conjugated anti-α7integrin (53-0010-05, AbLab). Cells were analyzed on a BD FACSCanto II apparatus (BD Biosciences).

### Histology and immunofluorescence analyses in mouse

Diaphragm and gastrocnemius muscles were frozen in nitrogen-chilled isopentane and kept at −80°C until use. Ten micrometers thick cryosections were prepared, permeabilized 10 min in 0.5% Triton X-100, and saturated in 2% BSA for 1 h at room temperature. Sections were incubated with primary rabbit anti-Laminin (L9393, Sigma Aldrich, 1:200), goat anti-Collagen I (131001, Biotech, 1:400), mouse anti-TGF-β1 (ab64715, Abcam, 1:200), or rat anti-CD64 (161002, BioLegend, 1:200) antibodies overnight at 4°C, then labeled with secondary FITC-conjugated donkey anti-rabbit, Cy3-conjugated donkey anti-goat, Cy3-conjugated donkey anti-mouse or Cy3-conjugated donkey anti-rat antibodies, respectively (Jackson Immunoresearch, 1:200). Fluorescent immunolabelings were recorded with a Zeiss Axio Scan.Z1 microscope connected to an ORCA-Flash4.0 V2 CMOS camera (Hamamatsu Photonics) at 20× magnification. Areas of Collagen1 were calculated with ImageJ software as previously described^22^ on 8–10 fields randomly chosen. Myofiber CSA was determined on whole muscle sections using Open-CSAM program as previously described.^55^

### Muscle force measurement

Mice were initially anesthetized in an induction chamber using 4% isoflurane. The right hindlimb was shaved before an electrode cream was applied at the knee and heel regions to optimize electrical stimulation. Each anesthetized mouse was placed supine in a cradle, allowing for a strict standardization of the animal positioning. Throughout a typical experiment, anesthesia was maintained by air inhalation through a facemask continuously supplied with 1.5%–2.5% isoflurane. The cradle also includes an electrical heating blanket in order to maintain the animal at a physiological temperature during anesthesia. Electrical stimuli were delivered through two electrodes located below the knee and the Achille’s tendon. The right foot was positioned and firmly immobilized through a rigid slipper on a pedal of an ergometer (NIMPHEA_Research, AII Biomedical SAS, Grenoble, France), allowing for the measurement of the force produced by the plantar flexor muscles (i.e., mainly the *gastrocnemius* muscle). The right knee was also firmly maintained using a rigid fixation in order to optimize isometric force recordings. Monophasic rectangular pulses of 0.2 ms were delivered using a constant-current stimulator (Digitimer DS7AH, Hertfordshire, UK, maximal voltage: 400V). The individual maximal current intensity was determined by progressively increasing the current intensity until there was no further peak twitch force increase. This intensity was then maintained to deliver stimulation trains at different stimulation frequency (i.e., ranging from 1 to 150 Hz). A resting period of at least 30 s was used between stimuli in order to avoid effects due to fatigue. For each stimulation train, isometric peak force was calculated. After a 3-min recovery period, force was assessed during a fatigue protocol consisting of 30 Hz stimulation trains of 0.3 s delivered once every second for 180 s. The peak force of each contraction was measured and averaged every five contractions. A fatigue index corresponding to the ratio between the last five and the first five contractions was determined. Force data were sampled at 1,000 Hz with a PowerLab8/35 (ADinstruments, Sydney, Australia) and analyzed with LabChart software (v8.1.17 ADInstruments, Sydney, Australia).

### Statistical analysis

All experiments were performed using at least three independent different cultures or animals except for Figures 3B–3G where two mice were used. Results are shown as mean ± SEM. Data normality was assessed using the Shapiro-Wilk test. Depending on the experimental, a two-tailed, unpaired Student’s t test or a one-way or two-way ANOVA with multiple comparisons correction was performed. In the case of non-normal data distribution, a two-tailed, unpaired Mann-Whitney’s U test or a Kruskal-Wallis test with multiple comparisons correction was performed. Statistical analyses were performed with GraphPad Prism version 9.0.0 (∗*p* < 0.05, ∗∗*p* < 0.01, and ∗∗∗*p* < 0.001).

## Data availability

The data that support the findings of this study are available from the corresponding authors upon reasonable request.

## Acknowledgments

This work was funded by grants from 10.13039/100014865Fondation Maladies Rares and AFM-Téléthon (N°POCs(2023)-121501) and 10.13039/501100001665Agence Nationale de la Recherche (ANR-19-CE14-0008 and ANR-18-CE18-0025-01). G.J. and A.K. were supported by AFM-Téléthon (MyoNeurALP 10.13039/100027925Alliance).

## Author contributions

Conceptualization, D.K., B.S., S.A., C.B., Y.C., J.G., B.C., R.M., G.L., and G.J.; methodology, I.A., A.G., M.R., A.K., S.B.L., F.T., A.F., M.M., J.S.B., and G.J.; analysis, I.A., A.K., S.B.L., F.T., A.F., J.S.B., J.G., and G.J.; writing—original draft, I.A. and G.J.; writing—review & editing, I.A., A.K., D.K., B.S., S.A., C.B., Y.C., J.G., B.C., R.M., G.L., and G.J.; supervision, J.G., B.C., R.M., G.L., and G.J.; funding acquisition, B.C., R.M., G.L., and G.J.

## Declaration of interests

The authors declare that they have no conflict of interest.

## Supplemental information

Document S1. Figures S1–S6 and Tables S1–S4

Document S2. Article plus supplemental information
