# Supplementary material for: Nanoparticle delivery of AMPK activator 991 prevents its toxicity and improves muscle homeostasis in Duchenne muscular dystrophy
Source: Mol Ther Methods Clin Dev. 2025 Aug 14;33(3):101564. doi: 10.1016/j.omtm.2025.101564 (PMC12433517; doi:10.1016/j.omtm.2025.101564)
Supplement: Document S1. Figures S1–S6 and Tables S1–S4 [file mmc1.pdf]

OMTM, Volume ■■

Supplemental information

Polymeric nanoparticle delivery of AMPK activator 991 prevents its toxicity and improves muscle homeostasis in Duchenne muscular dystrophy

Ilaria Andreana, Ananga Ghosh, Mathieu Repellin, Anita Kneppers, Sabrina Ben Larbi, Federica Tifni, Aurélie Fessard, Marion Martin, Jacqueline Sidi-Boumedine, David Kryza, Barbara Stella, Silvia Arpicco, Claire Bordes, Yves Chevalier, Julien Gondin, Bénédicte Chazaud, Rémi Mounier, Giovanna Lollo, and Gaëtan Juban

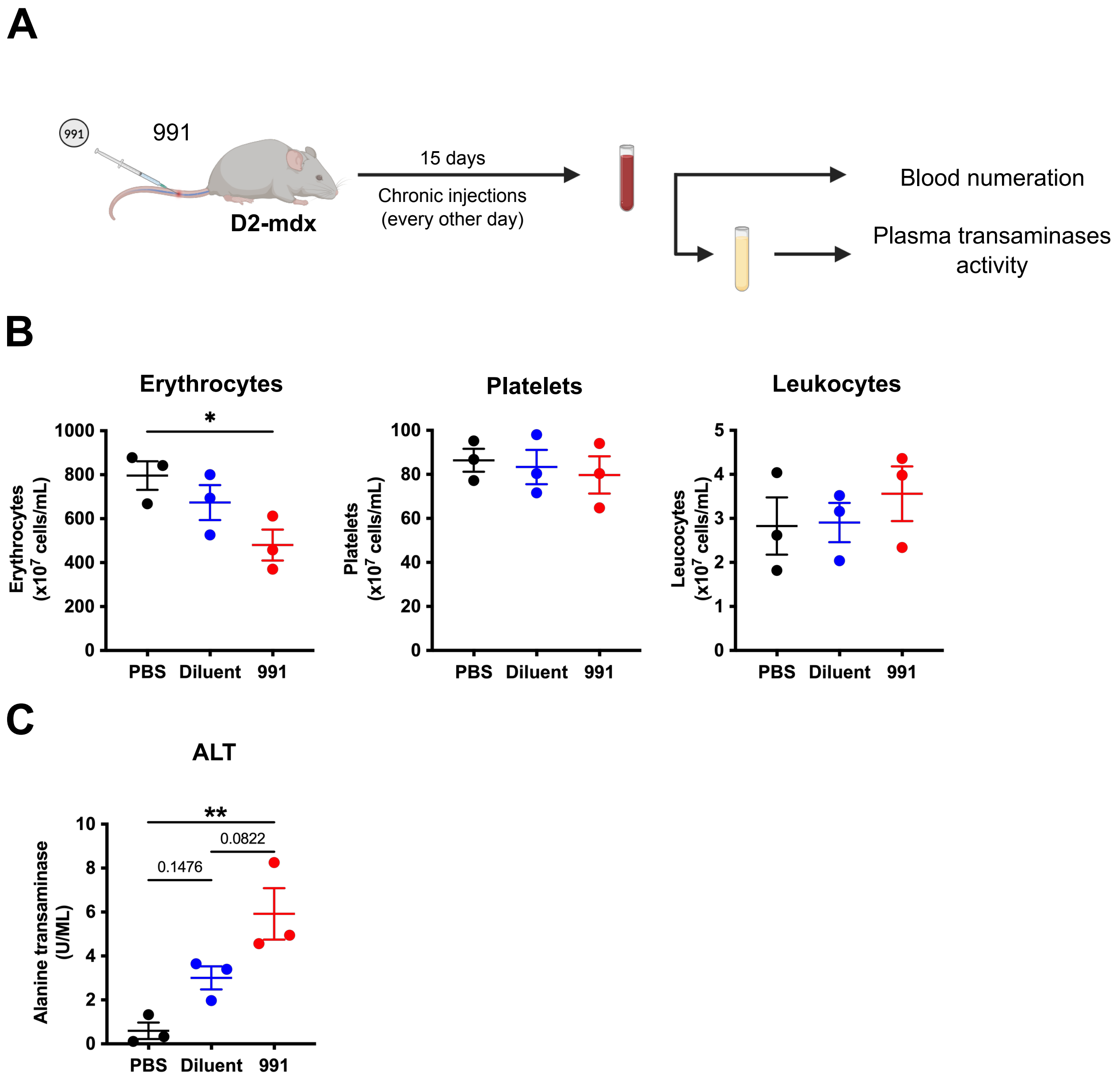
A
991
991
D2-mdx
15 days
Chronic injections
(every other day)
Blood numeration
Plasma transaminases activity
B
Erythrocytes
Erythrocytes (x10^7 cells/mL)
Platelets
Platelets (x10^7 cells/mL)
Leukocytes
Leucocytes (x10^7 cells/mL)
PBS Diluent 991
C
ALT
Alanine transaminase (U/ML)
0.1476
0.0822
PBS Diluent 991

Figure S1

Figure S1: **Chronic 991 treatment shows toxicity in vivo.** (A) D2-mdx mice were treated with chronic intravenous injections of PBS, Diluent or 991 for 15 days and blood collected. (B) Blood cell counts showing the concentration of erythrocytes, platelets and leukocytes. (C) Serum levels of alanine transaminase (ALT) enzyme. Results are shown as mean±s.e.m. of n=3 experiments. *p<0.05; **p<0.01 by one-way ANOVA with Tukey's multiple comparison correction.

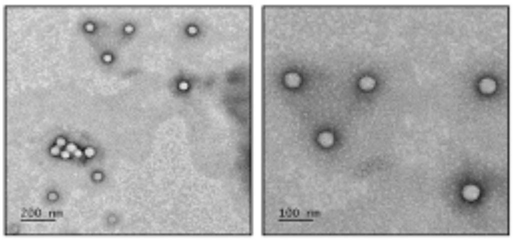

Figure S2

Figure S2: **Effect of freeze-drying on PLGA-991 NP morphology.** TEM images of freeze-dried 991-loaded PLGA NPs prepared by microfluidic technique after resuspension.

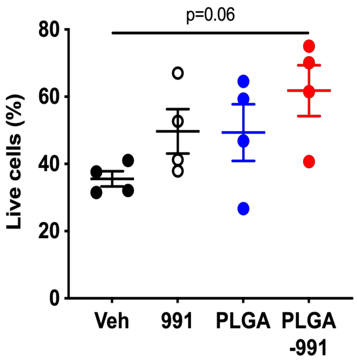

Figure S3

Figure S3: **Effect of PLGA-991 on macrophage viability *in vitro*.** BMDMs were polarized into fibrotic macrophages and treated with 20 µM of 991 alone, empty PLGA or PLGA-991 for 20 h and cell viability was assessed by flow cytometry after Annexin V and Hoechst labeling. The percentage of viable cells, identified as Annexin V^{neg} Hoechst^{neg}, is shown. Results are shown as mean±s.e.m. of n=4 experiments. One-way ANOVA with Tukey's multiple comparison correction was performed.

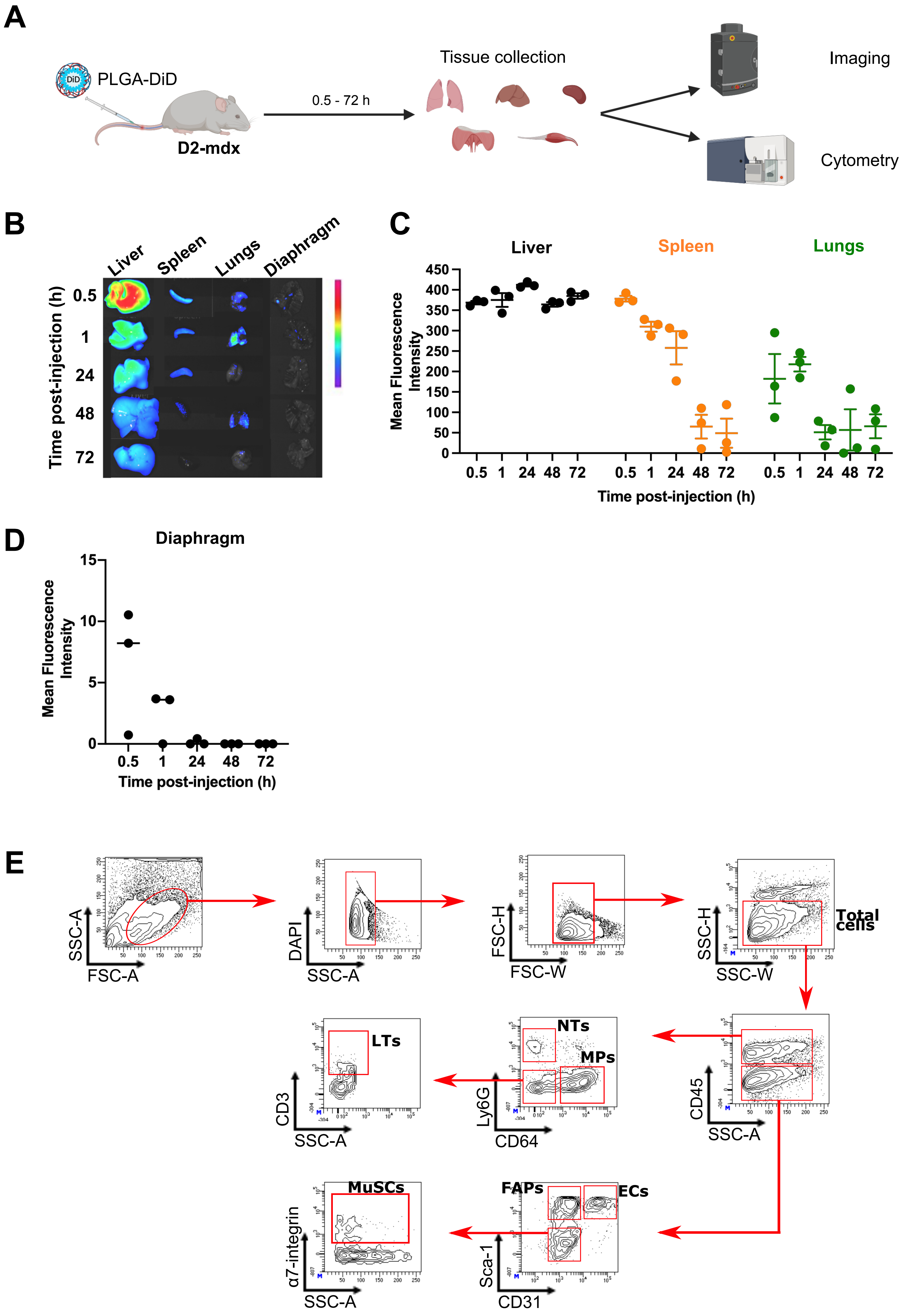
A
PLGA-DiD
D2-mdx
0.5 - 72 h
Tissue collection
Imaging
Cytometry
B
Liver
Spleen
Lungs
Diaphragm
Time post-injection (h)
0.5
1
24
48
72
C
Liver
Spleen
Lungs
Mean Fluorescence Intensity
Time post-injection (h)
D
Diaphragm
Mean Fluorescence Intensity
Time post-injection (h)
E
SSC-A
FSC-A
DAPI
FSC-H
FSC-W
SSC-H
SSC-W
Total cells
CD45
LTs
CD3
NTs
MPs
Ly6G
CD64
MuSCs
α7-integrin
FAPs
ECs
Sca-1
CD31

Figure S4

Figure S4: **PLGA NPs biodistribution in D2-mdx mice**. (A) D2-mdx mice were injected intravenously with DiD-loaded PLGA NPs and sacrificed 0.5, 1, 24, 48 and 72h later. (B-D) Organs were harvested and imaged to quantify DiD fluorescence. (B) Representative images of imaged organs. (C) Mean fluorescence intensity quantified in liver (black), spleen (orange), and lungs (green). (D) Mean fluorescence intensity quantified in the diaphragm muscle. (E) Gating strategy to discriminate muscle cell populations for the quantification of NP internalization by flow cytometry (Fig3). Shown are representative plots obtained from *Gastrocnemius* muscle.

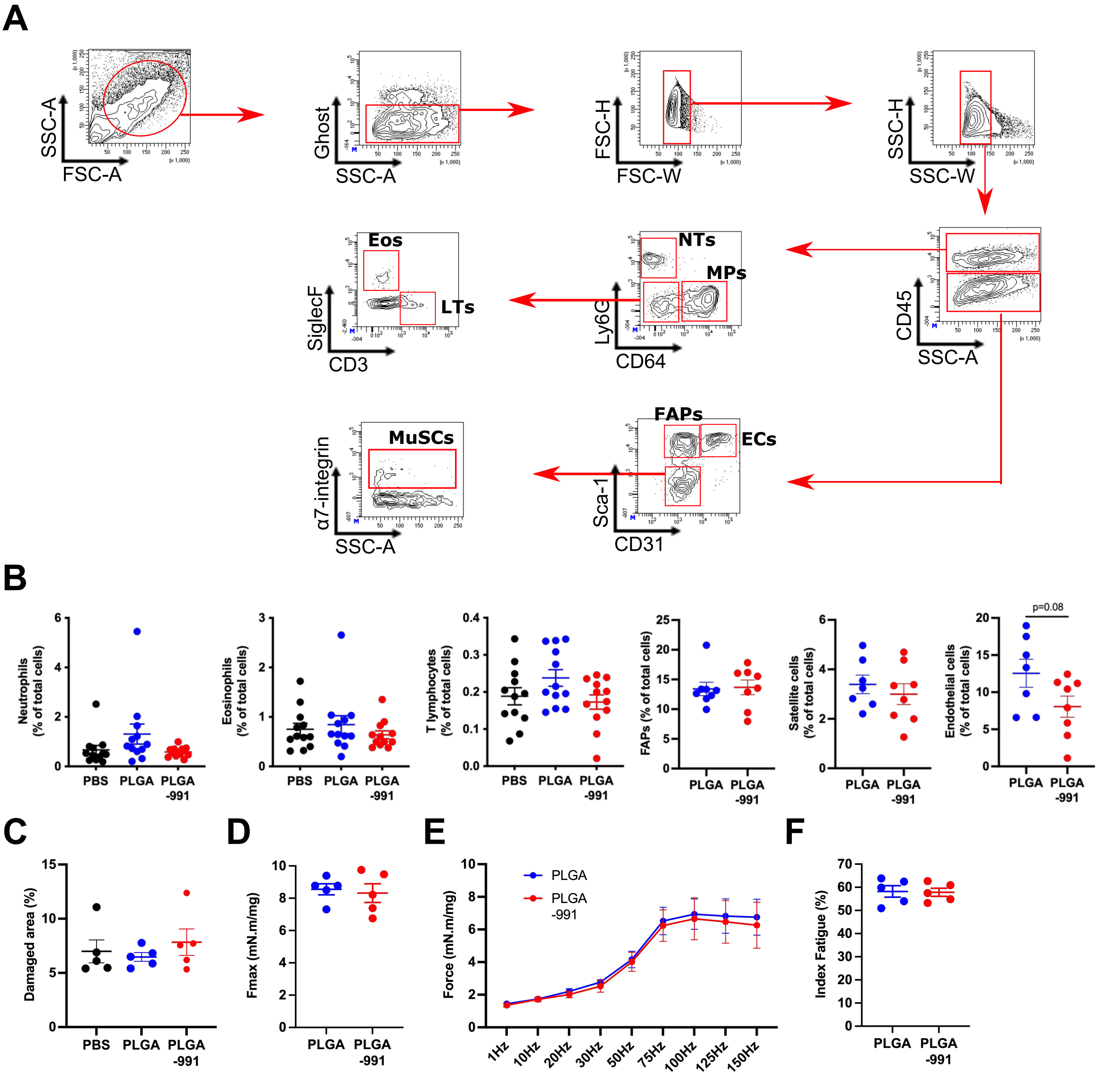
A
SSC-A
FSC-A
Ghost
SSC-A
FSC-H
FSC-W
SSC-H
SSC-W
CD45
SSC-A
NTs
MPs
Ly6G
CD64
Eos
LTs
SiglecF
CD3
FAPs
ECs
Sca-1
CD31
MuSCs
α7-integrin
SSC-A
B
Neutrophils (% of total cells)
Eosinophils (% of total cells)
T lymphocytes (% of total cells)
FAPs (% of total cells)
Satellite cells (% of total cells)
Endothelial cells (% of total cells)
p=0.08
PBS
PLGA
PLGA -991
C
Damaged area (%)
D
Fmax (mN.m/mg)
E
Force (mN.m/mg)
PLGA
PLGA -991
1Hz
10Hz
20Hz
30Hz
50Hz
75Hz
100Hz
125Hz
150Hz
F
Index Fatigue (%)

Figure S5

Figure S5: **Impact of PLGA-991 treatment on *Gastrocnemius*.** D2-mdx mice were treated with chronic intravenous injections of PBS, PLGA or PLGA-991 for 21 days as in Fig.5A, then *Gastrocnemius* was collected. (A) Flow cytometry gating strategy to discriminate cell types MPs: macrophages; NTs: neutrophils; LTs: T lymphocytes; Eos: eosinophils; FAPs: fibro-adipogenic progenitors; MuSCs: muscle stem cells; ECs: endothelial cells. Representative plots obtained on *Gastrocnemius* muscle from PBS-treated mice. (B) Quantification of neutrophils, eosinophils, T lymphocytes, FAPs, MuSCs and endothelial cells in the *Gastrocnemius*. (C) Percentage of damaged areas determined after IgG immunostaining. (D-E) *Gastrocnemius* muscle force production was measured. (D) Maximal specific force. (E) Specific force determined at different stimulation frequencies. (F) Fatigue index. Results are shown as mean±s.e.m. of n=5-12 experiments. Two-tailed unpaired Student's *t*-test (B "MuSCs" and "EC panels", D, F), two-tailed unpaired Mann-Whitney's U Test (B "FAPs" panel), one-way ANOVA with Tukey's multiple comparison correction (B "LTs" panel, C), or Kruskal-Wallis test with Dunn's multiple comparison correction (B "NTs" and "Eos" panels) were performed. In (E), two-way ANOVA with Tukey's multiple comparison correction was used.

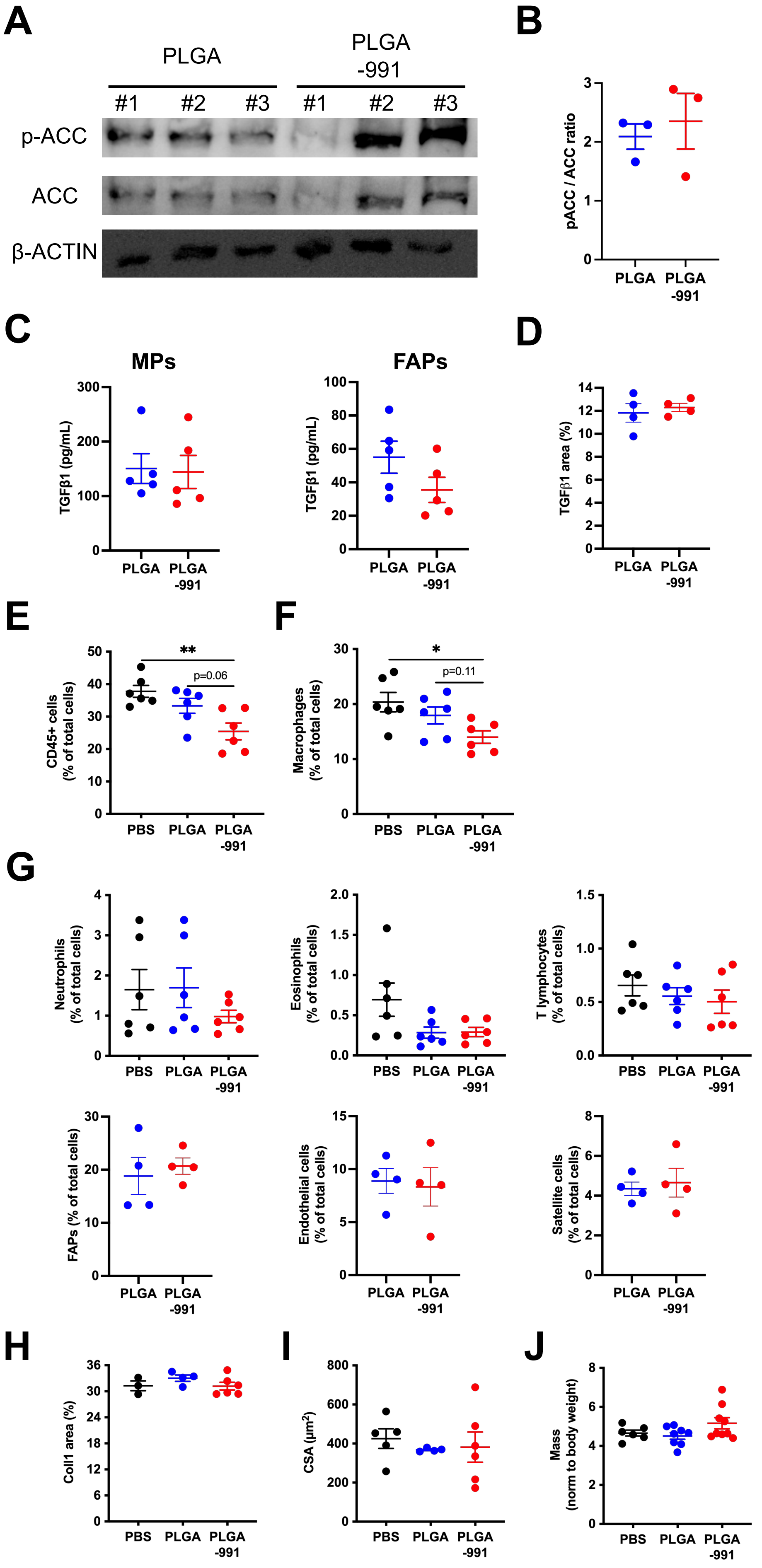
A
PLGA
PLGA -991
#1 #2 #3 #1 #2 #3
p-ACC
ACC
β-ACTIN
B
pACC / ACC ratio
PLGA
PLGA -991
C
MPs
FAPs
TGFβ1 (pg/mL)
D
TGFβ1 area (%)
E
CD45+ cells (% of total cells)
**
p=0.06
F
Macrophages (% of total cells)
*
p=0.11
PBS
G
Neutrophils (% of total cells)
Eosinophils (% of total cells)
T lymphocytes (% of total cells)
FAPs (% of total cells)
Endothelial cells (% of total cells)
Satellite cells (% of total cells)
H
Col1 area (%)
I
CSA (µm²)
J
Mass (norm to body weight)

Figure S6

Figure S6: **Impact of PLGA-991 treatment on diaphragm.** D2-mdx were treated with chronic intravenous injections of PBS, PLGA or PLGA-991 for 21 days as in Fig.5A, and diaphragm was collected. D2-mdx mice were treated with chronic intravenous injections of PBS, PLGA or PLGA-991 for 3 weeks, then diaphragm muscles were harvested. (A-B) The phosphorylation of ACC was quantified by immunoblot. (A) Representative immunoblots. (B) Quantification of the ratio between the phosphorylated over the total ACC proteins normalized to the β-ACTIN signal. (C) Macrophages (MPs) and fibro-adipogenic progenitors (FAPs) were isolated from diaphragm and their TGFβ1 secretion was determined by ELISA. (D) Percentage of TGFβ1 area determined after immunostaining on diaphragm cryosection. (E-F) Proportion of immune cells (CD45+) (E) and macrophages (F) determined by flow cytometry. (H) Percentage of Col1 area. (I) Mean myofiber cross sectional area (CSA) determined on Laminin immunolabeling. (J) Relative diaphragm muscle mass (in mg) normalized by body weight (in g). Results are shown as mean±s.e.m. of n=3-6 experiments. *p<0.05; **p<0.01 by two-tailed unpaired Student's *t*-test (B, C "MPs" panel, D, G), two-tailed Mann-Whitney's U test (C "FAPs" panel) or one-way ANOVA with Tukey's multiple comparison correction (E-J).

Table S1: Physicochemical characterization of blank and loaded PLGA NPs prepared by nanoprecipitation and microfluidic technique. Values are given as mean ± SD (n = 3). NPs: nanoparticles; PdI: polydispersity index; EE: Encapsulation Efficiency; DL: Drug Loading.

Formulation	Preparation method	Size (nm ± SD)	*PdI*	ζ (mV ± SD)	*EE* (% ± SD)	*DL* (% ± SD)
PLGA NPs	Nanoprecipitation	147 ± 3	0.05	−30.5 ± 0.4	/	/
991-PLGA NPs	Nanoprecipitation	169 ± 15	0.10	−46.4 ± 7.4	85.0 ± 12.0	10.9 ± 0.4
PLGA NPs	Microfluidic	70 ± 2	0.18	−50.1 ± 1.9	/	/
991-PLGA NPs	Microfluidic	81 ± 8	0.17	−49.1 ± 10	75.1 ± 7.8	9.4 ± 1.0

Table S2. Experimentation plan built according to a 2^3 full factorial design (runs 1 to 8) with two additional center points (runs 9 and 10) with the corresponding EE and DL responses (see legends in Table S2).

Run	PLGA (mg/mL)[a] (X_1)	*FRR* (v/v)[b] (X_2)	Drug (mg/mL)[c] (X_3)	Size (nm ± SD)	PdI	ζ (mV ± SD)	*EE*%	*DL*%
1	5	1:5	0.7	148 ± 11	0.28	−32.8 ± 4.2	100.0	8.9
2	10	1:5	0.7	70 ± 1	0.20	−43.8 ± 3.0	95.0	4.6
3	5	1:2	0.7	85 ± 6	0.11	−50.0 ± 3.7	50.3	6.1
4	10	1:2	0.7	90 ± 1	0.07	−50.5 ± 2.6	74.3	5.0
5	5	1:5	1.4	148 ± 1	0.16	−40.5 ± 0.5	78.1	18.3
6	10	1:5	1.4	110 ± 3	0.21	−47.1 ± 0.8	77.6	10.9
7	5	1:2	1.4	136 ± 1	0.14	−46.6 ± 0.1	75.0	13.6
8	10	1:2	1.4	110 ± 1	0.04	−43.8 ±2.5	86.4	9.5
9	7.5	1:3.5	1.1	129 ± 4	0.25	−43.4 ± 9.8	85.9	10.8
10	7.5	1:3.5	1.1	128 ± 3	0.25	−44.8 ± 0.2	92.5	11.3

[a] polymer concentration in the organic phase

[b] flow rate ratio (polymer-drug solution: aqueous phase)

[c] drug concentration in the organic phase

Table S3. Coefficients of synergistic models (Eq. 2) for EE and DL with their corresponding R^2 and experimental standard deviation SD_{exp}.

Coefficients	*EE* (%) (Y_1)	*DL* (%) (Y_2)
b_0	80.8	9.7
b_1	3.9	−2.1
b_2	−8.9	−1.1
b_3	0.1	3.5
b_{12}	5.5	0.8
b_{13}	9.8	−0.8
b_{23}	–	−0.5
R^2	0.930	0.986
SD_{exp}*	5.4%	0.8%

* standard deviation of the experimental error determined from repeated runs (degrees of freedom = 3)

Table S4. Physicochemical characterization of loaded-PLGA NPs prepared by microfluidic technique, before and after freeze-drying (n=3).

Formulation	Time	Size (nm ± SD)	PdI	ζ (mV ± SD)	Osmolarity
991-PLGA NPs	Before freeze-drying	81 ± 8	0.165	−49.1 ± 10	–
991-PLGA NPs	After freeze-drying	84 ± 18	0.190	−35.6 ± 2.9	0.286
